# Analysis of porcine body size variation using re-sequencing data of miniature and large pigs

**DOI:** 10.1186/s12864-018-5009-y

**Published:** 2018-09-19

**Authors:** C. Reimer, C.-J. Rubin, A. R. Sharifi, N.-T. Ha, S. Weigend, K.-H. Waldmann, O. Distl, S. D. Pant, M. Fredholm, M. Schlather, H. Simianer

**Affiliations:** 1 Animal Breeding and Genetics Group, Department of Animal Sciences, University of Goettingen, Albrecht-Thaer-Weg 3, 37075 Goettingen, Germany; 2 Science for Life Laboratory, Department of Medical Biochemistry and Microbiology, Uppsala University, Uppsala Biomedicinska centrum BMC, Husargatan 3, 75237 Uppsala, Sweden; 3 Institute of Farm Animal Genetics of the Friedrich-Loeffler-Institut, Höltystraße 10, 31535 Neustadt-Mariensee, Germany; 4 Clinic for Swine, Small Ruminants, Forensic Medicine and Ambulatory Service, University of Veterinary Medicine – Foundation, Bischofsholer Damm 15, 30173 Hannover, Germany; 5 Institute of Animal Breeding and Genetics, University of Veterinary Medicine – Foundation, Bünteweg 17p, 30559 Hannover, Germany; 6 Graham Centre for Agricultural Innovation, School of Animal & Veterinary Sciences, Charles Sturt University, Locked Bag 588, Boorooma St., Wagga Wagga, NSW Australia; 7 Department of Veterinary- and Animal Sciences, University of Copenhagen, Grønnegårdsvej 3, 1870 Frederiksberg C, Denmark; 8 School of Business Informatics and Mathematics, University of Mannheim, A5 6, 68131 Mannheim, Germany; 9 Center for Integrated Breeding Research, University of Goettingen, Albrecht-Thaer-Weg 3, 37075 Goettingen, Germany

**Keywords:** Goettingen Minipig, Whole genome resequencing, Body size, X-chromosomal QTL

## Abstract

**Background:**

Domestication has led to substantial phenotypic and genetic variation in domestic animals. In pigs, the size of so called minipigs differs by one order of magnitude compared to breeds of large body size. We used biallelic SNPs identified from re-sequencing data to compare various publicly available wild and domestic populations against two minipig breeds to gain better understanding of the genetic background of the extensive body size variation. We combined two complementary measures, expected heterozygosity and the composite likelihood ratio test implemented in “SweepFinder”, to identify signatures of selection in Minipigs. We intersected these sweep regions with a measure of differentiation, namely *F*_*ST*_, to remove regions of low variation across pigs. An extraordinary large sweep between 52 and 61 Mb on chromosome X was separately analyzed based on SNP-array data of F_2_ individuals from a cross of Goettingen Minipigs and large pigs.

**Results:**

Selective sweep analysis identified putative sweep regions for growth and subsequent gene annotation provided a comprehensive set of putative candidate genes. A long swept haplotype on chromosome X, descending from the Goettingen Minipig founders was associated with a reduction of adult body length by 3% in F_2_ cross-breds.

**Conclusion:**

The resulting set of genes in putative sweep regions implies that the genetic background of body size variation in pigs is polygenic rather than mono- or oligogenic. Identified genes suggest alterations in metabolic functions and a possible insulin resistance to contribute to miniaturization. A size QTL located within the sweep on chromosome X, with an estimated effect of 3% on body length, is comparable to the largest known in pigs or other species. The androgen receptor *AR*, previously known to influence pig performance and carcass traits, is the most obvious potential candidate gene within this region.

**Electronic supplementary material:**

The online version of this article (10.1186/s12864-018-5009-y) contains supplementary material, which is available to authorized users.

## Background

The livestock species of today display vast phenotypic variation. Domestication and breed formation processes have shaped these species by increasing the variation in traits related to, performance, fitness, morphology and appearance, thereby changing the - phenotypically rather uniform - wild ancestors to the illustrious collection of our modern breeds. Focusing on body size, Haldane [1] discussed a general principle as to why the horse is larger than the rabbit, or the cow is larger than the pig, and suggested that there must be a right size for a certain form of a body and a change in size must be accompanied with a change in form. In contradiction to that, a wide range of body size or weight is often seen within a single species, as for example in dogs [2]. Taking the example of pigs (*Sus scrofa*), the process of domestication of the wild boar led to animals that span from large fattening pigs to the so called ‘miniature pigs’ or simplified ‘minipigs’. Their sizes differ by up to one order of magnitude. Among the minipigs the Goettingen Minipig (GMP) is one of the smallest breeds under a stringent breeding scheme [3, 4]. The Goettingen Minipig is a composite breed developed in the 1960’s at the former Institute of Animal Breeding and Genetics at the Georg-August-University Göttingen in Germany. It was founded by crossing Minnesota Minipigs (MMP) with Vietnamese Potbellied Pigs (VPP). Later German Landrace pigs (LAR) were introduced to produce uniformly white animals [5]. This pig breed shows a form of miniaturization called “proportional dwarfism” which Simianer and Köhn [3] suggested to be a form of pituitary dwarfism, caused by lower secretion of growth hormones from the pituitary gland, leading to a decreased secretion of the insulin-like growth factor 1 (*IGF1*).

The availability of porcine SNP chips offers the possibility to screen the genome for regions carrying genetic variants associated with the reduced size of minipigs. Gaerke et al. [6] conducted a study on signatures of selection in GMP, MMP, VPP and LAR, using a 60 k SNP chip. They found that alleles from all founder breeds were still segregating in the GMP and identified numerous putatively positively selected regions in the GMP. They suggested that a pathway connecting *SOCS2* and *GRB10* with *IGF1* could exist that plays an important role in the dwarfism of the GMP. Due to the limited marker density of the SNP array it was not possible to reveal causal mutations.

The current reference genome is based on the sequence of a Duroc pig with the first studies, using this reference to provide insight into the porcine demography and evolution [7] and into the patterns that domestication and anthropogenic selection have left in the porcine genome [8], which were published in 2012. While these studies used diverse sets of pig breeds from all over the world, minipigs were not included. The very same month, the genome of a highly inbred Chinese Wuzhishan minipig was published [9] as an additional reference genome for Asian pigs, which have been domesticated independently from European pigs [10]. The present study aims at comparing WGS data of a diverse set of pig breeds to unveil the genetic mechanisms behind body size variation, and more specifically the miniaturization in pigs. In working towards this aim, we compared a group of miniature pig breeds to a group of large pig breeds by screening for highly differentiated regions under selection in the minipigs. Such candidate regions were subsequently screened for candidate genes with a putative effect on growth or body size, and the postulated effects on body-size of one of the identified candidate region was confirmed with data of an independent crossbreeding experiment.

## Results

### Number of SNPs

Biallelic SNPs are the most common class of variants used in genetic studies of animal genomes. Since SNP calling from WGS data is not limited to prior knowledge on variant positions, the number of SNPs is an indicator of variability in the analysed dataset and of strictness of the variant discovery and filtering. SNP calling from the DNA sequencing data revealed 46 × 10^6^ biallelic SNPs genome-wide, of which 29 × 10^6^ were polymorphic or fixed for the alternative allele in the minipigs. After filtering, 35 × 10^6^ loci were polymorphic in all samples and 19.8 × 10^6^ only in minipigs. For European domestics, European wild boars, Asian domestics and Asian wild boars, these numbers were 19 × 10^6^, 9.4 × 10^6^, 19.5 × 10^6^ and 19.2 × 10^6^, respectively. Subsequent in-silico pooling left 27.6 × 10^6^ loci with sufficient information to compare minipigs against large pigs.

### Phylogeny

**Fig. 1:**
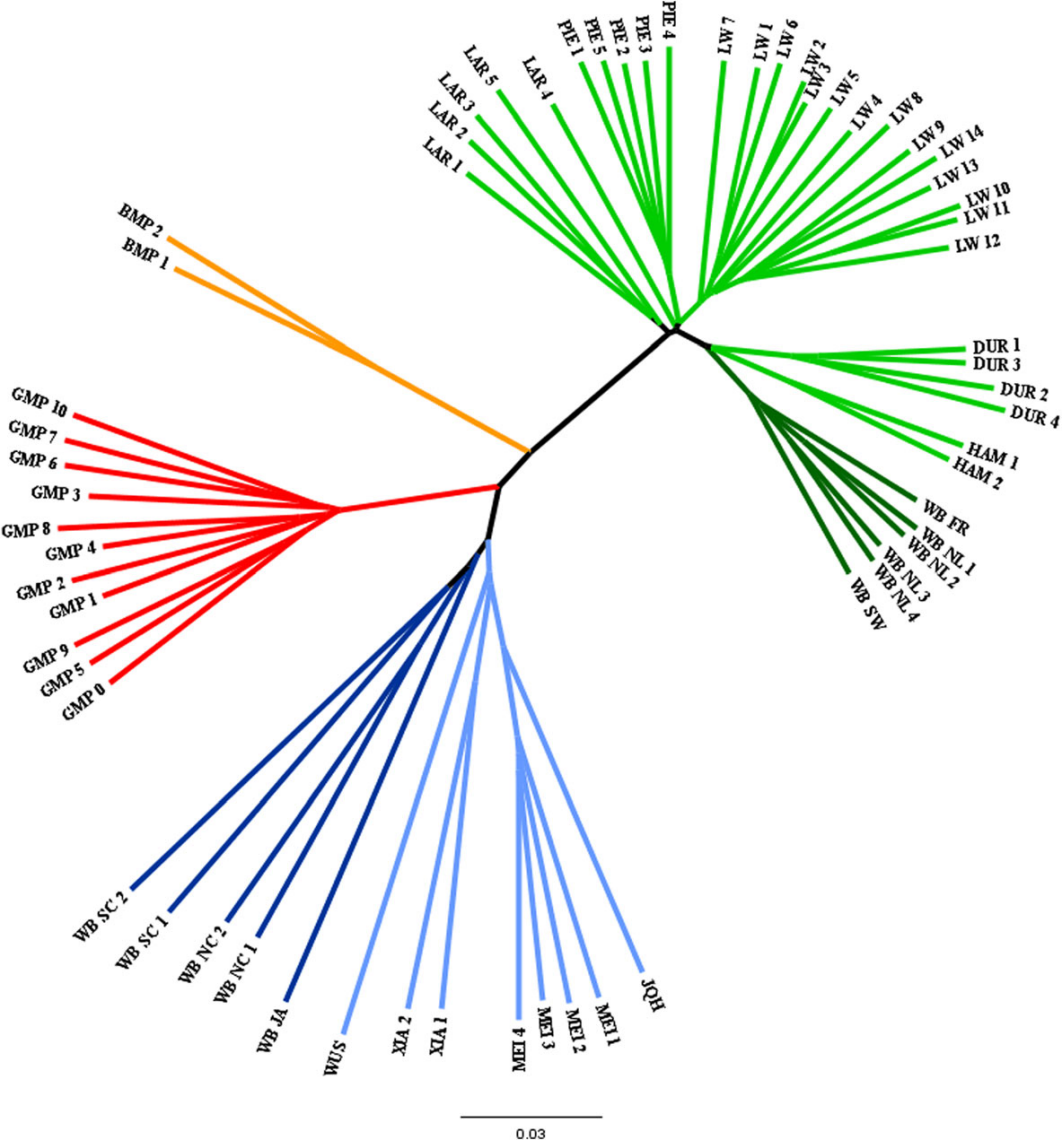
Neighbor-joining tree computed from pairwise IBS distances. Based on SNP data of the randomly selected chromosomes 1, 8 and 13 for all individuals (due to computational limitations). Asian wild boars in dark blue, Asian domestics in light blue, European wild boars in dark green, European domestics in light green, Mini-LEWE in orange and Goettingen Minipigs in red

When comparing large pig breeds to minipigs, it is important to account for stratification within each contrasting group to ensure, that no breed specific signals will be identified. The analysis of genetic distances between sampled breeds revealed a clear division of European and Asian large pigs, with minipigs clustering closer to the Asian pigs (Fig. 1; see also MDS in Additional file 1). Estimation of *F*_*ST*_ also showed that the minipigs were closer to the Asian breeds than to the European breeds (*F*_*ST*_ = 0.08 and 0.12, respectively), while both minipig breeds were marginally closer to the domestic groups of both continents than to the respective wild boars. This effect is smaller for the GMP (GMP to European domestic/ wild: 0.14, 0.16; GMP to Asian domestic/ wild: 0.10, 0.11), whereas there is clear distinction for the BMP, which is much closer to both domestic groups than to the wild boars (BMP to European domestic/ wild: 0.07, 0.14; BMP to Asian domestic/ wild: 0.08, 0.11). The *F*_*ST*_ value between both minipig groups is 0.09. The highest overall differentiation has been estimated between European and Asian wild boars (additional information in Additional file 2 and Additional file 3).

### Selective sweeps

**Fig. 2:**
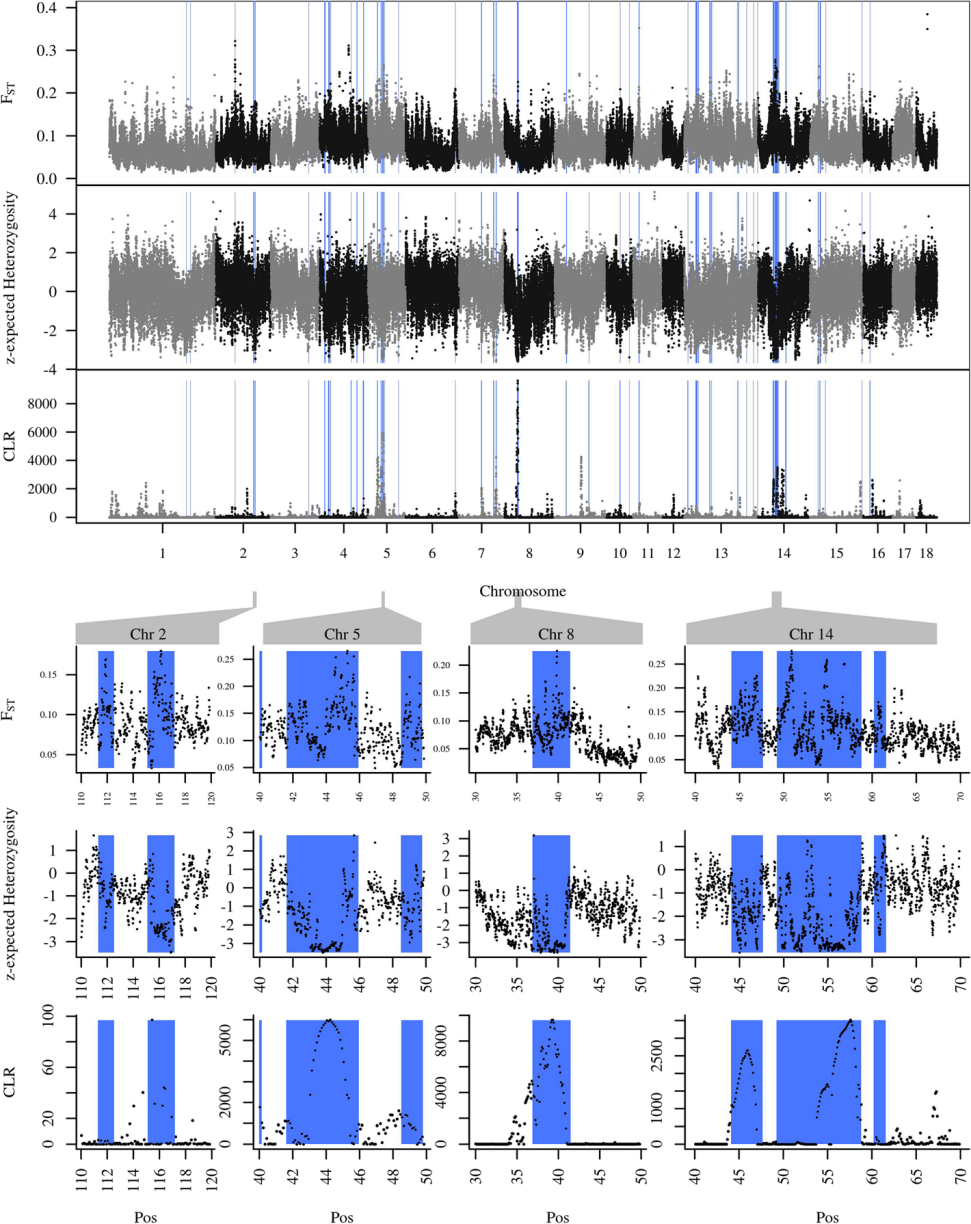
CLR test and normalized expected heterozygosity within minipigs and F_ST_ between large pigs and minipigs. Regions on chromosomes 2, 5, 8 and 14 identified as putative selective sweeps are highlighted; Blue rectangles underlie detected putative sweeps

We searched for genomic regions under selective pressure for body size using a so-called selective sweep analysis, employing a combination of decreased expected heterozygosity, SweepFinder and F_ST,_ and subsequently identified candidate genes within these regions. The selective sweep analysis revealed considerable parts of the genome as putatively being targeted by selection for growth. Not every chromosome was affected equally. Most of the 49 identified signals extended between 1 Mb and 2.5 Mb, but one on chromosome 14 reached nearly 10 Mb. The other large signals were located on chromosomes 5 (2.8 and 4.3 Mb), 8 (4.6 Mb), 13 (5.2 and 2.9 Mb), 14 (3.6 Mb) (Fig. 2) and chromosome X (48 Mb; not shown). SweepFinder detected fewer, but larger regions, whereas the regions detected by decreased heterozygosity were more numerous but smaller. The exceptionally large region on chromosome 14 consists of an accumulation of many small signals reflecting reduced heterozygosity and two large signals from SweepFinder. The union of both signals gives a nearly uninterrupted huge selective sweep signal.

### Genes in sweeps and gene ontology over-representation

All genes within sweep regions were annotated and gene ontologies (GO’s), which represent functional categories, linked to every detected gene were checked for over-representation of certain GOs within sweeps compared to the unselected background, to identify functional categories rather than single candidates. The Ensembl porcine gene set 79 annotation within sweep regions on the autosomes revealed 524 genes (Additional file 4), belonging to 2006 unique gene-ontology terms. 55 of these gene ontologies were found to be overrepresented within sweeps by using a Fisher’s exact test *p*-value lower than the 5% quantile threshold of the empirical distribution function for the respective ontology. Table 1 shows a selection of gene ontologies over-represented in putative sweeps (see also Additional file 5).

**Table 1:** Selected gene ontologies over-represented in putative sweeps

No.	Fisher-P	Empirical *p*-value	Number of genes in term and sweep	Fold Enr.	GO Term Name
1	0.0017	0.0002	7	3.94	Z disc
2	0.0012	0.0014	4	0.26	negative regulation of transcription from RNA polymerase II promoter
3	0.0050	0.0040	5	4.38	protein tyrosine/serine/threonine phosphatase activity
4	0.0172	0.0059	4	3.94	Microvillus
5	0.0060	0.0063	4	5.25	regulation of alternative mRNA splicing, via spliceosome
6	0.0149	0.0067	13	1.99	mitochondrial inner membrane
7	0.0033	0.0067	10	2.75	protein dephosphorylation
8	0.0024	0.0078	54	1.52	Mitochondrion
9	0.0096	0.0081	3	6.44	leukocyte tethering or rolling
10	0.0125	0.012	3	5.91	ventricular cardiac muscle cell action potential
22	0.0272	0.0222	10	2.13	actin cytoskeleton
25	0.0101	0.0239	2	11.81	mitochondrial electron transport, ubiquinol to cytochrome c
27	0.0101	0.0248	2	11.81	positive regulation of growth
31	0.006	0.0286	4	5.25	social behavior

A literature review for all genes belonging to the overrepresented GO terms with a focus on properties characterizing minipigs revealed a comprehensive set of genes with interesting putative functions (Table 2). Among them are genes like *COMT* and *PATZ1* with direct effects on growth or size in other organisms, *ACOT4* and *PKP2*, which are involved in growth factor signaling, or genes directly linked to growth in swine, for example *PPARG* that is suspected to be a key factor in porcine growth, conformation and fatness. Additionally, we found a considerable number of genes with links to the MAPK signaling cascade, e.g. *MAPK1* and *PTPRR*, involved in glucose and lipid metabolism, or putatively responsible for insulin resistance or diabetes type II or obesity.

**Table 2:** Candidate genes from potentially enriched ontologies with putative functional link to minipigs

Gene name	Function	Reference
*ACACB*	Downregulated by *TGFB1*; influencing type-II-diabetes; obesity and lipid metabolism	Zhou et al. [87]; Ma et al. [88]
*ACOT4*	Linked to *FGF21 in mice*	Muise et al. [89]
*ADAMTS12*	Blocks Ras*/* MAPK pathway	Llamazares et al. [90]
*COMT*	Reduced birth weight in humans	Sata et al. [33]
*DUSP28*	Activator of MAPK pathway	Wang et al. [91]
*HYAL1, HYAL2*	Overexpressed in the placenta of the smallest pig fetuses	Vallet et al. [92]
*LTBP1*	*TGFB* signaling, role in the regulation of human height	Lango Allen et al. [31]
*MAGOH*	Influences MAPK	Roignant and Treisman [93]
*MAPK1*	Coding central proteins *ERK2* in the Ras*/* MAPK	Reviewed by Cobb et al. [94]
*NDUFB9*	Severe growth-hormone deficiency	Riedl et al. [95]
*OSM*	Diabetes type II	Sanchez-Infantes et al. [96]
*PATZ1*	Null mice were retarded in growth, Homozygote animals were 10 to 20% smaller, than their litter mates of the same sex	Valentino et al. [34]
*PKP2*	Associates *EGF*	Kazlauskas [97]
*PPARG*	Muscle specific expression; deletion causes insulin resistance in mice; key role in pig growth; reduced size in pre-pubertal children	Crooks et al. [43]; Hevener et al. [42]; Puig-Oliveras et al. [44]; Cecil et al. [45]
*PRKAR2A*	Obesity and lipid metabolism	Park et al. 2012 [98]
*PTPRR*	Member of the MAPK pathway	Hendriks et al. [99]
*SOD1*	Depressor of the MAPK pathway central genes *ERK1/2*	Juarez et al. [100]

### Strong selective sweep on chromosome X

**Fig. 3:**
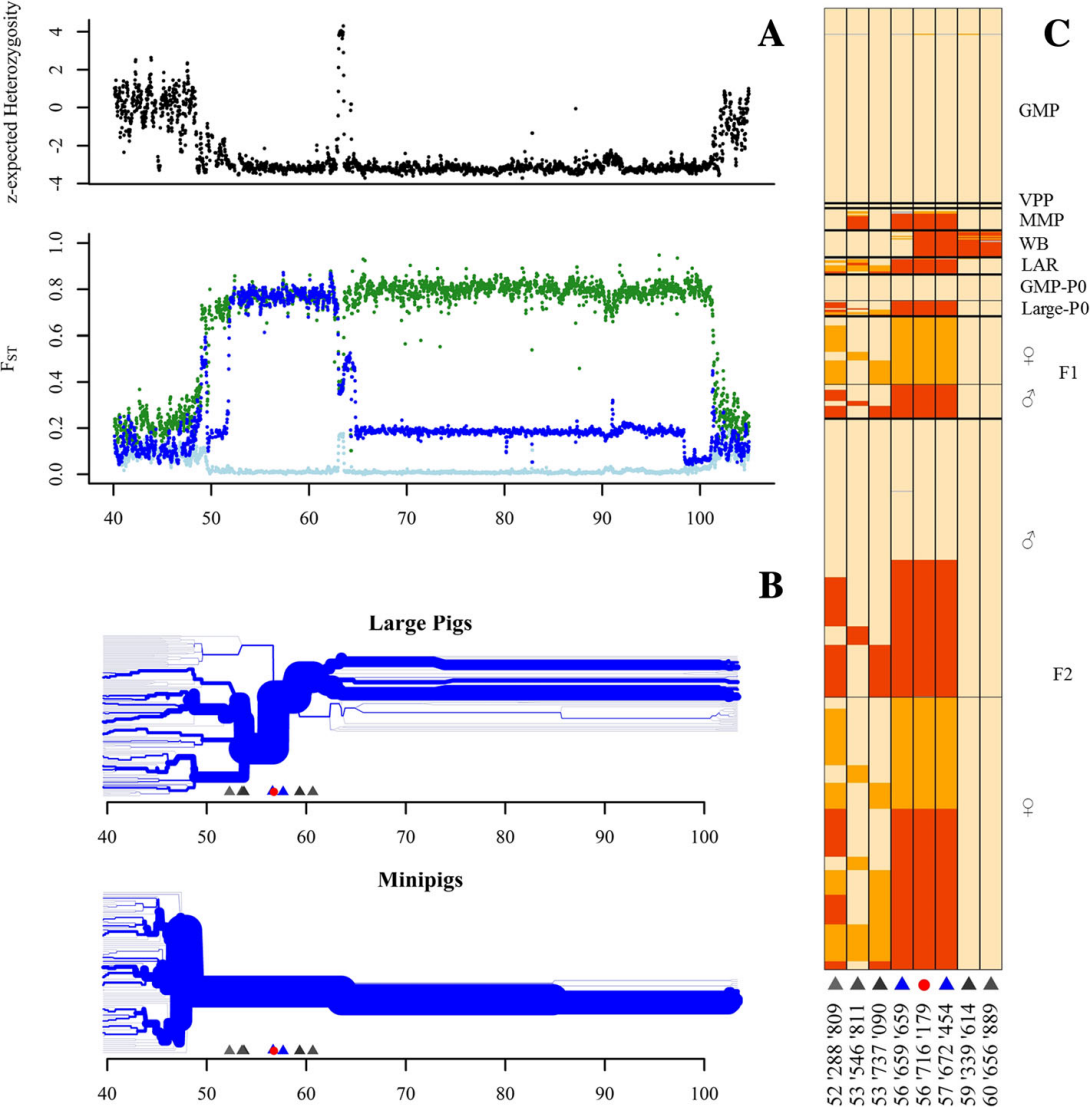
Large X-chromosomal sweep region, linkage decay and co-located genotypes in cross-bred animals. **a** Normalized expected heterozygosity in minipigs and fixation index between minipigs and European (green), South Asian (lightblued) and North Asian (darkblue) across the critical region of Chromosome X; **b** Haplotype breakdown within the major sweep region in all large pig breeds and in the minipig breed respectively, positions in Mb, centered at 56′716’179 Mb; **c** Allelic state at 8 analyzed SNPs in the sweep region between 50 and 62 Mb (red = homozygous for minipig allele, orange = hemi−/ heterozygous, beige = homozygous for opposite allele), positions in bp. Red dot and blue and grey triangles indicate SNP positions; (♀ = female, ♂ = male)

The major selective sweep on chromosome X (chr. X) known from the studies of Rubin et al. [8] and Ai et al. [11] is also found in the minipigs. It is known that this sweep consists of two majorly un-recombining haplotypes of about 9 and 39 Mb, respectively. Figure 3a shows a substantial decrease of the expected heterozygosity within the minipigs in a 48 Mb region in the middle of chromosome X between 52 Mb and 100 Mb. The fixation index shows that this region consists of two separate sub-regions. The first part, approximately inside the interval 52 Mb to 61 Mb, is typical for the minipigs and shared with South Asian pigs, whereas differentiation in the second part implies that the minipigs are also similar to some breeds from North Asia. We postulated that this genomic region might have an effect on body size and therefore utilized data of a former cross-breeding experiment, to estimate QTL effects for each existent haplotype.

**Fig. 4:**
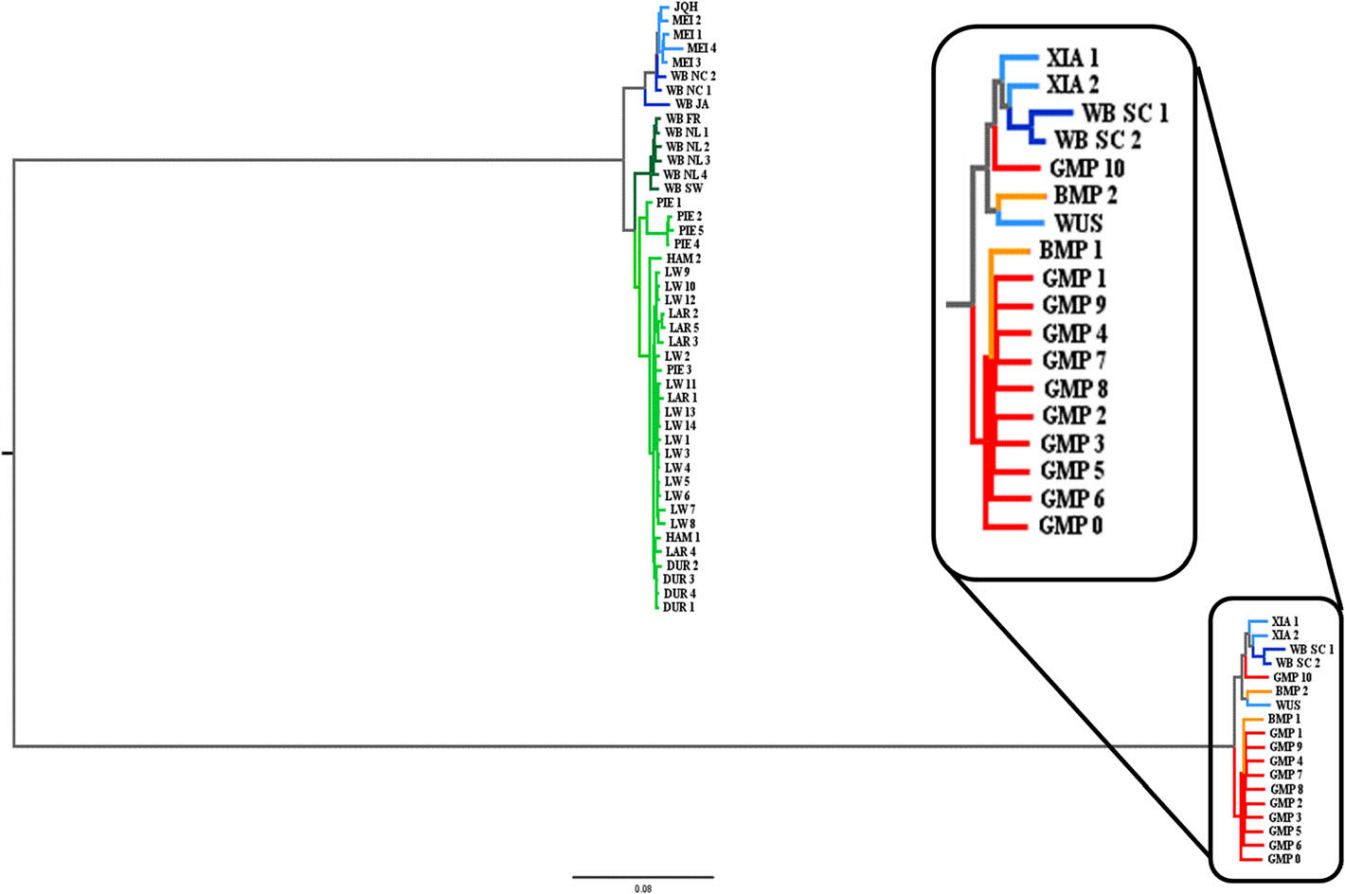
Neighbor-joining tree for all markers between 52 and 61 Mb on chromosome X. Asian wild boars in dark blue, Asian domestics in light blue, European wild boars in dark green, European domestics in light green, Mini-LEWE in orange and Goettingen Minipigs in red

The phylogenetic tree of all sequenced animals based on all markers inside the first region (Fig. 4), shows that the haplotype carried by the minipigs is shared with the Xiang pigs and two wild boars from South China. The sub-tree for the second region clusters the samples into two main groups, the first comprising the minipigs, the Xiang, the Meishan, the Jiangquhai and the South Chinese wild boars, and the second all European breeds and the wild boars from North China and Japan (Additional file 6).

### Analysis of SNP chip data

Since the haplotype carried in the region chrX:52–61 Mb appears to be typical for minipigs, we used genotyping data from two former studies [6, 12] to determine the haplotypic state of animals with recorded phenotypes in order to enable the estimation of the effect of the minipig haplotype on size. The Illumina PorcineSNP60 BeadChip contains 23 SNPs located on chromosome X between 52 and 61 Mb according to the current map based on the genome build 10.2. Filtering removed 7 individuals for poor genotyping (call rate < 10%), 3 SNPs that were missing and 13 SNPs, which had a low minor allele frequency. 8 SNPs (Additional file 7) passed the filtering, three of them in the beginning of the region around 53 Mb (MARC0056564, MARC0046345, H3GA0051807), three in the center around 57 Mb (INRA0056742, H3GA0051810, MARC0013223) and two at the end around 60 Mb (INRA0056744, H3GA0051814). At the first three loci, all minipigs are homozygous for a guanine-cytosin-guanin haplotype, while also two Duroc females from the Danish study are found being heterozygous for this haplotype. Therefore, these three markers are not informative to determine the origin of the respective allele in the cross-breds. The genotypes at the three center loci perfectly coincide with the affiliation of a pig to the large pigs or the minipigs, respectively (Fig. 3c). We only observed heterozygous genotypes in animals from the cross-breeding experiment. Thus, these markers are fully informative to decide whether a cross-bred animal carries the large pig haplotype or the minipig (South Asian) haplotype in the interval between 52 and 61 Mb. The two markers at the end of the interval are homozygous in most European wild boars. Omitting the markers in the beginning of the interval, there are only three clearly distinguishable haplotypes within the sampled breeds in the first region of the selective sweep. Figure 3b shows the LD decay, depicted as a bifurcation diagram centered at position 56′716’179 for both, the large pig haplotype, based on all SNP array genotypes of all large pigs without wild boars and the minipig derived haplotype without Minnesota Minipigs. The minipig derived haplotype is stable over the whole first part of the selective sweep and is barely variable in the second part. The large pig haplotype is less stable and it splits up within the borders of the first sweep region and in the beginning of the second sweep region. The distribution of the haplotypes can be found in Additional file 8.

### Inheritance of the haplotypes in cross-bred animals

Under the assumption of no recombination within the selective sweep region on X and the cross-breeding scheme of Pant et al. [12], we expected a certain distribution of combinations of these haplotypes in animals of the F_1_ and F_2_ generation. Using the aforementioned SNP loci, we determined which haplotypes were inherited. As shown in Table 3, all F_1_ females should be heterozygous and all males should be hemizygous for the large pig haplotype. In the F_2_, half of the females are expected to be homozygous for the large pig haplotype, the other half heterozygous. The F_2_ males should be hemizygous, one half for the minipig haplotype, the other half for the large pig haplotype. The observed haplotypes match the expected Mendelian proportions.

**Table 3:**
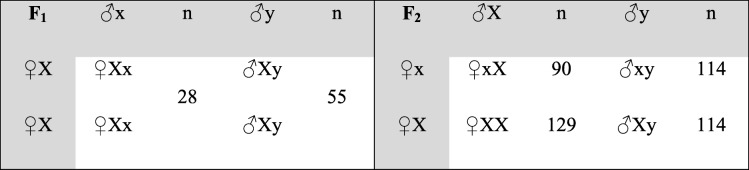
Theoretical inheritance of the two segregating haplotypes on the X- chromosomes in the cross-bred pigs

Capital and low case letters indicate whether a haplotype is originating from a large pig or a minipig founder animal, respectively. Numbers of animals with the respective haplotype constellation are shown in columns right of each haplotype

### Effect estimators of linear models

The distribution of phenotypic values of the analyzed traits of height and length at the ages of scanning and slaughtering are displayed in Table 4.

**Table 4:** Sample size, average age, means and standard deviations for the analyzed traits in F_2_ cross-breds

Trait	N	Age [days]	Mean [cm]	SD [cm]
Height at scanning	432	63 (45–166)	39.93 ± 0.21	4.39
Height at slaughter	263	242 (166–439)	65.30 ± 0.31	5.05
Length at scanning	432	63 (45–166)	48.56 ± 0.28	5.91
Length at slaughter	410	242 (166–439)	84.16 ± 0.31	6.21

**Fig. 5:**
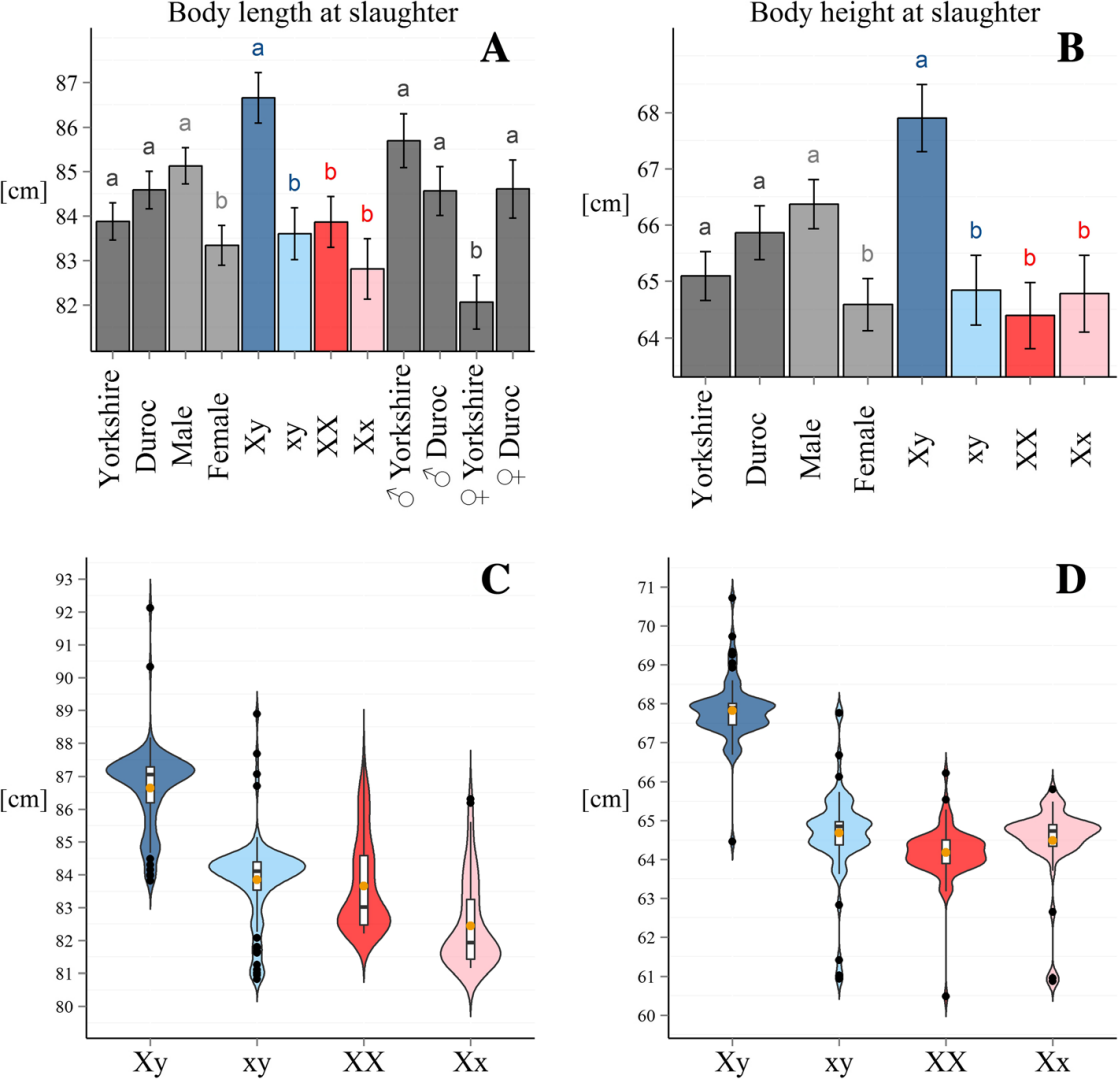
Estimated effects of the X-chromosomal haplotype state on body size. **a** and **b** Least-square means for the significant effects for traits length and height at slaughter. **c** and **d** Violin plots of phenotypes, corrected for all significant covariates, apart from haplotype/ sex for the respective traits

Table 5 shows the covariates considered in the final models for the analysis of the different traits. All non-significant higher interactions were removed from the model. We could not find a significant influence of the haplotype on the length at age of scanning and height at age of scanning, although in the latter, the *p*-value was 0.0718 and the subsequent conservative LSD test showed significant differences between the haplotypes. Only the sex and the age were important for length at age of scanning. The breed of the mother in the P_0_ did not influence the size traits of young animals at age of scanning. Figure 5 shows the estimated effects of the inherited X-chromosomal haplotype on the traits “height at slaughter” and “length at slaughter”.

**Table 5:** Factors with significant influence on growth traits

Trait	Breed	Sex	Age	Age^2^	Haplotype(Sex)	Breed* sex	Breed*Age	Breed*Age^2^
Length at age of scanning		0.003	0.016	< 0.0001				
Height at age of scanning		0.29	0.057	< 0.0001	0.072			
Length at age of slaughter	0.102	0.004	0.008		0.0004	0.003	0.060	
Height at age of slaughter	0.038	0.005	0.593	0.543	0.0014		0.031	0.025

For the two traits, where the haplotype effect was significant, males carrying an X-chromosome copy inherited from the minipig ancestor were significantly smaller than the ones carrying the large pig copy, while there was no significant size difference between homozygous females of large pig origin and the heterozygous females.

The respective violin plots of the linear predictors, which can be interpreted as corrected phenotypes for the four haplotype*sex classes, show a clear distinction of the males by haplotype origin.

### Genes inside the sweep

We found 18 known genes lying within the first sweep region on chromosome X (Additional file 9). One of those is the androgen receptor gene *AR*, which has several functions in physiological processes related to growth, body conformation and reproduction. Besides its crucial role in spermatogenesis and male fertility [13, 14], it is involved in spinal muscle atrophy [15], bone growth [16] and in the determination of body size in humans, where a mild supply of testosterone to boys of under-average size stimulated growth and sexual development without compromising final height [17]. It is located in a large QTL for pig performance and carcass traits [18] and has been identified as a candidate underlying this QTL [19]. Mice with a knock-out of the *AR* suffer a late onset of obesity while being normally sensitive to insulin [20]. Additionally *AR* is activated by the growth factors *IGF*, *KGF* and *EGF* in tumors [21].

## Discussion

This is the first study using whole genome resequencing to discover signatures of selection for body size comparing minipigs against individual and pool data of various pig breeds. Direct comparison of contrast, made up from various pig breeds each, mainly differentiated in body size only, appeared as a powerful approach to determine the genetic background of growth and size in minipigs. The high informational density of the next-generation-sequencing data promised deeper insights as the array based approaches before.

### SNP calling

One of the often discussed issues for the quality of WGS studies is the quality of the alignment and the depth at which samples have been sequenced. The proportion of aligned reads to the current reference genome of a Duroc [7] was roughly 90% for GMP and 87% for BMP, similar to the proportion we find in European and Asian domestics and confirms Frantz et al. [22] findings when mapping the WGS data of Asian wild boars (*Sus verrucosus)* against the Duroc reference. When the de novo assembled GMP genome with a length of 2.44 Gb [23] was mapped against the Duroc reference of 2.3 Gb, about 96% could be placed on chromosomes. Therefore, using the Duroc reference genome to evaluate minipig genomes appears appropriate, although there is an inherent risk of missing out important parts of the genome.

The number of discovered SNPs in a genome depends on the sequence identity between the reference genome and the examined samples, which is in turn dependent on the phylogenetic distance, the variation inside the breeds and the number of individuals. Additionally, the reliability of calling SNPs and determining genotypes from WGS data is also dependent on the sequencing depth, where for example, reliable calling of a homozygous (heterozygous) SNP requires 15X (30X) coverage [24]. From this point of view, the coverage of all minipigs was sufficient for SNP detection, but proper genotype assignment could be improved by resequencing at higher depth.

### Phylogeny

Analysis of the genetic distance and F_ST_ of the sequenced animals showed a clear differentiation between European and Asian pig breeds. This result is in agreement with the current scientific consensus, that domestication occurred independently in Europe and Asia around 9000 years ago [10]. In contrast to European breeds which evolved straight from the wild boar [25], the history of Asian pigs is more complex: After dispersal into the islands and Oceania, interrupted by feral states, pigs were eventually transferred to the Asian mainland [26]. Later, the Chinese populations diverged into a northern and a southern strain [11]. Our results confirm the gap between south Chinese (Xiang, Wuzhishan) and north Chinese domestic breeds (Jiangquhai, Meishan) but appear less clear than in Ai et al. [11].

In the phylogenetic tree, the Goettingen and the Mini-LEWE are located between the Asian and the European cluster. Looking at the breed histories, both breeds are synthetic crosses of the Vietnamese Potbellied Pig with European breeds. In the case of the Mini-LEWE, the crossing partner was the Saddleback pig and “Deutsches veredeltes Landschwein” (comparable to Large White) [27]. The GMP was established using German Landrace and the Minnesota Minipig [5], itself a cross bred of five breeds of not completely resolved but mostly north American feral, possibly Asian origin [28]. This might be the reason for the BMP being closer to the European cluster than the GMP.

### Signatures of selection

#### Polygenic effect of autosomal genes on growth

This study compares two contrasting groups in order to reveal the genetic background of the reduced body size: various large pig breeds from all over the world versus a group of two minipig breeds. Such a study design has been proven efficient before in detecting regions of differentiating selection before in chicken [29] and pigs [8] and has revealed comprehensive sets of candidate genes in both studies. Although it is known that low recombination rates in combination with inbreeding have the potential to produce signatures similar to selective sweeps [30], the inclusion of two genetically highly diverged minipig breeds should attenuate this problem. We discovered numerous putative sweep regions containing a comprehensive gene set and a first conclusion could, therefore, be that the genetic background of size differentiation is rather polygenic than mono- or oligo genic. This is not surprising, since it is known for other vertebrate species like humans [31] and chicken [32] that growth has a polygenic background. The consecutive analysis of over-representation for the respective gene ontologies provided a similar picture. A variety of ontologies reached significance, comprising ontologies with functions related to growth traits and energy metabolism, like “mitochondrion” and “positive regulation of growth”. The most potentially enriched ontology was ‘Z disc’, referring to a structural element of the muscle. The overrepresentation of genes related to mitochondria suggests that the energy metabolism might be a key element for growth restriction in minipigs. Some of the genes in enriched ontologies are known to have direct effects on growth and size development or even dwarfism: A *COMT* variant increases the risk of having children with reduced birth weight [33], knock out of *TPST2* or *PATZ1* leads to growth retardation in mice [34, 35].

A former study by Gaerke et al. [6] on the same GMP stock using a 60 k SNP array came to similar results. They also discovered numerous regions under putative selection comprising several genes with known effect on growth and suggested a pathway connecting *SOCS2*, *GRB10* and *IGF1* as potential cause of small body size in minipigs. This finding supported the hypothesis of Simianer and Köhn [3], that the minipig experiences a form of pituitary dwarfism, comparable to Shetland pony and Dexter cattle, supposedly caused by a deficiency of *IGF1*. This hypotheses seems natural, since the effect of *IGF1* on growth in, for example, Pygmies [36] is known for long. In case of a mutation in an *IGF* gene, a signature of selection would be expected around the respective gene as it was found in dogs, where small breeds carry a unique coding sequence of *IGF1* [37]. However, using WGS data, we did not observe striking signals of selection near any of the known *IGF* genes or receptor loci. This coincides with findings of Zenobi et al. [38] who concluded that the size difference between normal sized and minipigs is neither related to serum levels of *IGF1* or *IGF2*, nor to a missing response to or reduced secretion of growth hormones. Reduced transcription, manifested in low transcription levels of the *IGF* genes or other growth hormones, could be ruled out and alterations in the underlying genes seem unlikely. But still the insulin growth factor signaling cascade is a widely considered key mechanism for growth. Our results suggest an alternative function: A possible mechanism behind the dwarf phenotype could be a resistance of the target tissues to insulin. Symptoms of this, i.e. a hampered blood glucose clearance after insulin stimulation, which could be facilitated by a disordered lipid metabolism [39] or an intrauterine growth restriction [40] have been found in a feeding trial with Goettingen Minipigs [41]. Focusing on the breeds used in the cross-breeding for GMPs, the Vietnamese Potbellied Pig was the smallest, but also the most obese one [5]. Even after generations of closed breeding, the major part of the GMP genome can be attributed to the VPP [6], suspected to be the origin for the genetically determined tendency to obesity of current GMPs. The detected signatures of selection contained genes either directly influencing insulin resistance or traits such as obesity or muscle fiber composition. Among these genes *PPARG* is an outstanding candidate, having direct effects on insulin resistance [42] and muscle fibers [43]. Furthermore its effect on growth has been proven before in humans and pigs [44, 45].

Another kind of proportional dwarfism is caused by growth hormone (*GH*) deficiency [46] which resembles the phenotype of the “Laron dwarfism”, that is accompanied by severe growth retardation and obesity [47]. *GH* is also secreted in the pituitary gland and it was recently communicated that a knock-out of the growth hormone receptor gene *GHR* using genome editing technology led to further miniaturization of a bama minipig at 15 kg maturity weight [48]. However, focusing on genes belonging to *GH* or its receptor genes, we find only the CLR test to show increased evidence of selection about 1 Mb away from *GHR*, but no sign of differentiation between the large and the minipig group. Therefore our results do not support the hypothesis that selection on one of the *GH* genes is underlying the minipig dwarfism.

Among the detected genes are also genes known to be involved in the mitogen activated protein kinase pathway (MAPK) that controls cell proliferation and differentiation. Klingseisen and Jackson [49] report that this pathway plays a prominent role in growth processes and in the primordial dwarfism. This form of dwarfism leads to a proportional growth restriction causing a phenotype similar to the pituitary dwarfism. Besides others, we found the central gene of the Ras/MAPK pathway *MAPK1* in one of the largest sweep regions and *MAPKAPK3*, located in a large sweep on chromosome 13, which is known to be involved in the mediation of growth inhibiting signals [50] and has been found differentially expressed in the pituitary gland between the large and miniature strain of the Diannan pig [51].

#### Major effect of the X chromosomal sweep

The porcine X-chromosome carries a selective sweep of outstanding extent [8]. Using the Chinese Wuzhishan genome reference, Ai et al. [11] located this region of 48 Mb within the borders of 44 to 91.5 Mb, which corresponds to the region 52 to 100 Mb on the Duroc reference that we identified as a selective sweep exhibiting low expected heterozygosity in minipigs. We conclude from the same size of the region, the inclusion of partly the same samples in both studies and the nearly completely conserved haplotypes in our SNP chip analysis, that these two regions are analogous to each other. A sweep of comparable physical size was not found in recent selection signature studies in horse [52], sheep [53], chicken [29], dogs [54] or rabbits [55], suggesting that this region might carry vital genetic variations kept together due to haplotype effects or that recombination in the region is suppressed. Ai et al. [11] found a recombination breakpoint between a 14 Mb and a 34 Mb stretch, leading to three major groups of haplotypes, a European, a Southern Chinese and a Northern Chinese recombined haplotype. They explained the high differentiation of these three haplotypes with an introgression from a common ancestor even before domestication followed by a strong selective pressure for habitats in high altitudes. They concluded that this large region remained consistent over long time, since the estimated low recombination rate in this region could facilitate larger sweeps [56]. They speculated that the reason for decreased variation was an enrichment of poly(T) sequences leading to a reduced recombination rate as known from human genomes [57]. Using the Duroc reference for the analysis of our resequencing data, we find that the minipigs carry a haplotype similar to the South Asian samples we employed. This haplotype could be identified as the formerly known southern Chinese haplotype [11], based on the Wuzhishan samples considered in both studies. The SNP chip data within the first sweep region (52–61 Mb) shows that the founder breeds must have provided both the European and the South Asian haplotype into the GMP during breed establishment: The Vietnamese Potbellied Pig carries the South Chinese haplotype, while the Landrace carries the haplotype found in European wild boars and the Minnesota Minipigs carries both haplotypes. Thus it is surprising that we can solely detect the South Chinese haplotype in our current GMP stock, suggesting that the European haplotype must have disappeared during breed consolidation. Since the GMP was always selected for small size and high fertility, these two traits might underlie the selection against the European haplotype.

The subsequent evaluation of an F2 generation from a GMP x Yorkshire and a GMP x Duroc cross for four body size traits showed that males inheriting the GMP haplotype were significantly smaller than a male carrying the European haplotype for three of the four traits, while there was no significant effect on the fourth trait (Length at scanning). These results confirm that the analyzed region carries an allele influencing body size. Due to the cross-breeding scheme no females carrying only the minipig haplotypes on both chromosomes were available. The lack of a significant differentiation between females carrying the large pig haplotype on both copies of the chromosome X and heterozygous females indicates that the large pig haplotype could carry an allele that is dominant over the allele of the minipig haplotype covering the effect of the GMP allele, even though another study [58] found that the respective minipig allele of the androgen receptor AR, located in this haplotype, was dominant over a Duroc derived copy It also could be due to the mosaic nature of the X-chromosomal activation pattern in female eutherians [59]. At the single cell level, half of the body cells are deemed to carry either an active copy of the large haplotype or the GMP haplotype. Therefore, cells carrying the large pig haplotype might attenuate the size decreasing effect of the cells carrying the GMP copy.

The differences of 3.5% (3 cm) in body length at the age of slaughtering and 4.4% (3 cm) in height at age of slaughtering are QTL effects of considerable magnitude. Reviewing other QTL studies on size, height and growth shows that the underlying QTL architecture can be highly different dependent on trait or organism. Whereas, in humans, height is a highly heritable trait, influenced by at minimum 180 genetic loci [31] and SNP effects explain up to 45% of the phenotypic variance [60], only a small portion could be attributed to QTLs. Gudbjartsson et al. [61] identified 27 QTL explaining only 3.7% of the population variance in height, composed of single effects of about 0.3 to 0.6 cm, which was confirmed by other studies (Visscher [62]: 0.4 to 0.8 cm average effect size for a QTL; Hirschhorn and Lettre [63]: 0.3 to 0.6 cm effect on adult height). In domestic animals, larger QTL effects have been found. Signer-Hasler et al. [64] reported that two QTL together explain 18.2% of the heritable genetic variation in horses (~ 0.5 cm and ~ 1 cm for height at withers, respectively). They suggest the higher efficiency of QTL studies in domestic animals compared to humans to be due to the existence of isolated populations with reduced heterogeneity. In a cattle cross breeding scheme, a QTL next to *PLAG1*, *CHCHD7* and *MOS* was found with an allele substitution effect of 2 cm height at withers [65]. Rubin et al. [8] found that genotype combinations at two loci, *LCORL* and *PLAG1*, together explained a difference of 5.3 cm in body length in domestic pigs. Since we could not find signals of selection neither at *LCORL* nor *PLAG1* in our study, it is noticeable that the effect size of the chrX locus described herein has a similar effect size. Among the genes located in this region, the androgen receptor appears to be the most prominent candidate for a gene underlying the growth differences between pigs carrying opposite haplotypes. *AR* is influenced by several growth factors [21], has known function in growth processes [16, 17] and underlies the obesity phenotype that is commonly found in minipigs [20, 66]. Another study on the effect of the *AR* [19] on performance and carcass traits based on a cross-breeding experiment made up with Duroc and MiniLEWE also found that Duroc and MiniLEWE carry different copies of the *AR*. It could be shown that the MiniLEWE allele led to higher expression of the *AR* in several tissues including the uterus, and had effect on several performance and carcass traits. The haplotype that contains *AR* carried by all studied GMP was most likely identical to the aforementioned MiniLEWE allele and introduced by the Vietnamese Potbellied Pig during breed foundation. This pig breed was originally not only chosen for its small size, but also for the much higher litter size compared to the Minnesota Minipig [5]. Since there is a correlation between body size and litter size in mammals [67], which Ferguson et al. [68] estimated to be *r* = 0.2 in pigs, the current breeding scheme for low body weight and high fertility might have favored the Asian haplotype and *AR* seems to be one of the underlying causal genes for the miniature body size.

## Conclusion

Comparison of WGS data of minipigs against data of various large pig breeds is a logical approach in order to reveal the genetic background of body size in pigs. Signature of selection analysis with multiple complementary methods provided a comprehensive set of putative sweep regions, spanning approximately 2% of the autosomal genome. The set of associated genes and the consecutive GO term overrepresentation analysis suggest that energy metabolism, alterations in central elements of the MAPK pathway, and a possible insulin resistance might play a role in body size inheritance of miniature pigs. Additionally, the density of resequencing data proved to be especially useful to analyze a large sweep region on chromosome X, since the SNP chip available so far holds just few SNPs of limited information in that region. We identified three SNPs on the genotyping chip, serving as perfect markers to determine the respective haplotypic state of an individual in future studies. The effect size of the QTL of 3 cm in body length and height underlying this selective sweep is comparable to the largest QTL for body size traits known from other studies in mammals.

## Methods

### Analysis of whole genome resequencing data

#### Samples and raw data preparation

We extracted DNA from 10 representative contemporary GMP sows from the experimental herd of the University of Goettingen. DNA from 2 Mini-LEWE (BMP) sows, a miniature breed developed in Berlin, Germany and a DNA-pool of 10 female BMPs from the University of Veterinary Medicine Hannover was also prepared. Whole genome re-sequencing was performed at the Science for Life laboratory at Uppsala University, Sweden on an Illumina HighSeq2000 as paired end sequencing with an aimed average sequencing depth of 12X. The raw sequencing data is deposited in ENA under project accession PRJEB27654.

We added Publicly available re-sequencing data underlying the studies of Rubin et al. [8], Fang et al. [9] and Vamathevan et al. [23]. These samples contained breeds of Asian and European origin, both domestic and wild, and comprised animals of the breeds Duroc (DUR), Hampshire (HAM), Jiangquhai (JQH), Large White (LW), Landrace (LAR), Meishan (MEI), Pietrain (PIE), Xiang (XIA), European wild boars (WB FR, WB NL, WB SW), Asian wild boars (WB SC, WB NC, WB JA), a Wuzhishan (WUS) and one Goettingen Minipig (GMP) (Additional file 10).

We aligned all sequence reads to the reference genome susScrofa3 (build 10.2; [7]) using the Burrows-Wheeler algorithm as implemented in the software bwa [69]. The read trimming parameter was set to q = 5. We then sorted the alignments with Samtools [70] and used Picard tools [71] to mark duplicates without removal, to down-sample the data of the single Goettingen Minipig to a coverage comparable to the other minipig individuals and to construct indices for the alignments. Single Nucleotide Polymorphisms (SNPs) were called with GATKs Unified Genotyper [72, 73].

In order to obtain a reliable dataset for the selective sweep analysis, we applied a stringent filtering process on the variant call set, by first removing InDels and multi-allelic SNPs and filtering with GATK for a comprehensive set of quality criteria described in the methods section. The filters for chromosome X were adjusted separately taking into account the reduced depth of this chromosome in males. In addition, to keep a sample record a minimum genotype quality (GQ) of 20 was required for sequenced individuals and a minimum depth of coverage of 4 was required for pools.

#### In-silico pooling

For further analyses we constructed two contrasting in-silico pools: the large pig virtual pool (LPP) made up of Duroc, Hampshire, Jiangquhai, Large White, Landrace, Meishan, Pietrain, the European wild boars and the Asian wild boars. The minipig *in-silico* pool (MPP) comprised the Goettingen Minipigs, the Mini-LEWE and the Mini-LEWE-pool. For this, we calculated the reference allele frequency per breed for each locus. For each called SNP, the reference allele frequency in each *in-silico* pool was then calculated as the unweighted average of the respective breed reference allele frequencies. SNP loci for which less than 50% of the breeds in one of the two groups had a record were excluded.

#### Selection signature detection

For the detection of genomic regions with influence on the small size of the minipigs, we calculated heterozygosity and *F*_*ST*_ with custom R scripts [74] and combined it with the composite likelihood ratio (CLR) test, implemented in SweepFinder [75].

We calculated expected heterozygosity per locus as *H*_*exp*_ = 2*p*(1 − *p*) where *p* is the reference allele frequency in the MPP and afterwards averaged it in sliding windows of 100 kb with 80% overlap. We then normalized the *H*_*exp*_values of individual windows into Z-scores by adjusting the value using the mean and standard deviation derived from all 100 kb windows along autosomes and the X-chromosome independently. We defined candidate selective sweeps using an outlier approach whereby a window that fell below a value of − 2.34 (lowest 1%) was required to initially call a sweep, such sweeps were then extended to each side until values exceeded − 1.64 (lowest 5%).

The CLR test [75], implemented in SweepFinder was applied to the same 100 kb windows of the filtered SNP data of all individuals belonging to the Goettingen Minipigs and the Mini-LEWE. We excluded invariable loci across both groups and took the highest 1% of the signals further analysis.

The differentiation between the LPP and the MPP was determined by the fixation index

$$ {F}_{ST}=\frac{\sum {n}_i{\left({p}_i-\overline{p}\right)}^2/\Big(2\overline{n\Big)}}{\overline{p}\left(1-\overline{p}\right)} $$

altered after [76], where *p*_*i*_ is the frequency of the reference allele in group *i*, $\overline{p}$ is the weighted mean frequency of the first allele in both groups, *n*_*i*_ is the number of samples within a group *i*, and $\overline{n}$ is the average group size. We averaged the values across the same windows of 100 kb with 80% overlap as for heterozygosity and detected regions of increased differentiation by the same method as used for expected heterozygosity, with the highest 1% and 5% of the actual values taken as thresholds.

**Fig. 6:**
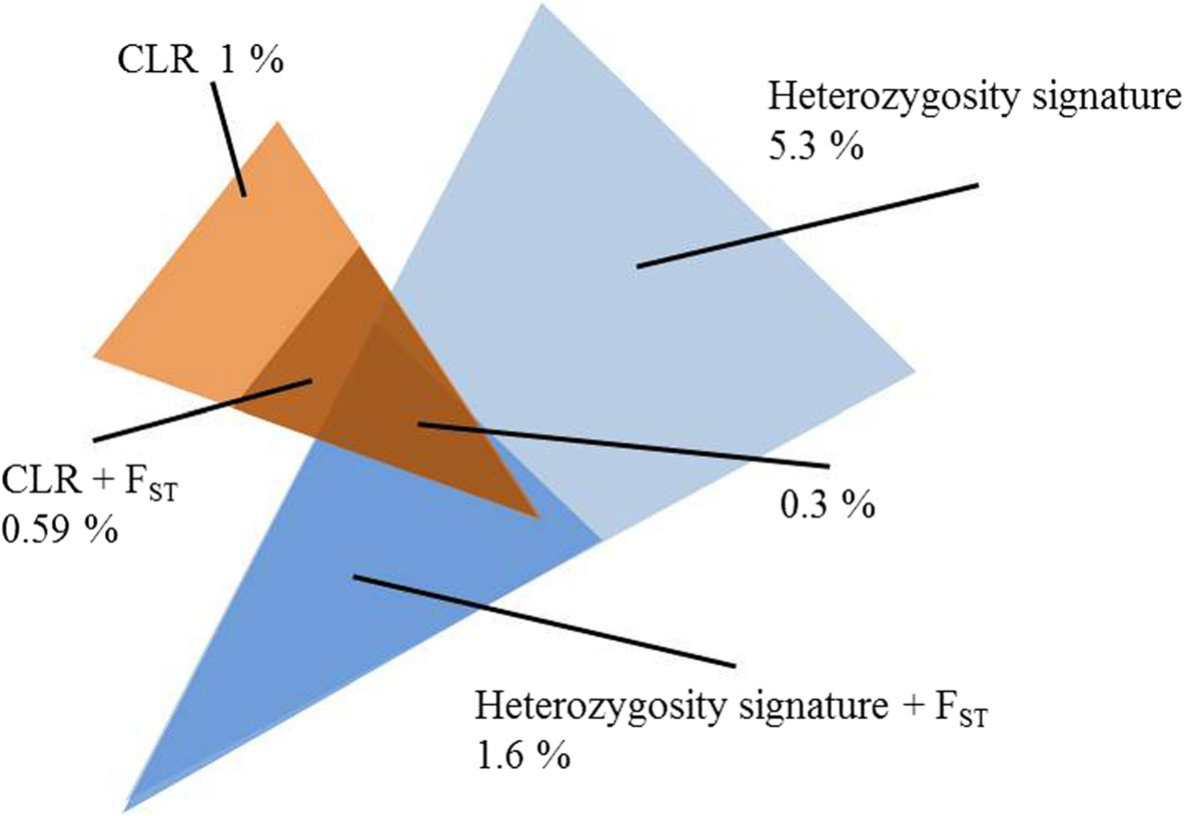
Overlaps between selection signature detection methods

A selective sweep was assumed, when regions showed decreased expected heterozygosity in the minipig or when the composite likelihood ratio test overlapped with signals of high differentiation between the two groups. We required a minimum width of 200 kb and extended the final regions by 0.5 Mb to each side. Figure 6 shows the proportions of the autosomes detected to be under putative selective pressure. The CLR test detected 1% of the genome as putative sweeps of which 59% intersected with outstanding F_ST_ signals. The heterozygosity signature method found 5.3% of the genome to be under selection, ~ 30% thereof (1.6% of the genome) intersecting with extreme F_ST_ signals. 0.3% of the whole genome was shared between CLR and heterozygosity signature. Finally, we used the union of CLR and expected Heterozygosity signals intersecting with F_ST_ for further analysis.

#### Phylogeny

We constructed phylogenetic trees from biallelic SNPs, extracted from filtered VCF-files with VCFtools [77] and from array data with customized R-code. We calculated the pairwise distance with Plink [78] as *1 - similarity between samples*, where similarity was the proportion of a genome of an individual being identical by state (IBS) with another animal’s genome. Based on this, we constructed the neighbor joining tree using PHYLIP [79]. We estimated pairwise *F*_*ST*_ values from all autosomal SNP loci with 90% or more animals with genotypes that passed filters. This was done in each contrasting combination of the individual data of European breeds, Asian breeds and minipigs and for the subgroups European domestic breeds, European wild boars, Asian domestics, Asian wild boars, Goettingen Minipigs and Mini-LEWE, respectively (additional information on the groups can be found in Additional file 2 and Additional file 3). Subsequently, we averaged values at all loci to gain a genome wide *F*_*ST*_ value.

#### Gene annotation and gene overrepresentation analysis

We annotated genes within regions of interest using the Ensembl Pig Gene set 79 [80] and, subsequently, conducted a gene ontology (GO) overrepresentation analysis by using Fisher’s exact test [81]. We calculated fold enrichment *FE* as

$$ FE=\frac{a}{\frac{\left(a+b\right)\left(a+c\right)}{a+b+c+d}}, $$

with *a* being the number of genes within a sweep and the respective gene ontology, *b* being the number of genes within a sweep but outside the respective gene ontology, *c* being the number of genes in a respective gene ontology but outside a sweep and *d* being the number of genes outside a sweep and outside the respective gene ontology [82, 83]. All statistics were applied on all GO terms for which genes had been found within a putative selective sweep. To account for possible bias resulting from assumption violations of the Fisher’s exact test (e.g. independency of the genes) as well as the multiple testing problem, we conducted a permutation analysis to construct empirical significance thresholds for the calculated *p*-values. To this end, we shifted the set of sweep regions along the genome by a random number of base pairs between 1 and the genome length, while retaining sweep sizes. Genes were then annotated to the shifted set of sweep regions and Fisher’s exact test *p*-value was re-estimated for each ontology term found in our original annotation. This random shifting should assure the resulting *p*-values to reflect the case when the null hypothesis is true. Based on 5000 replications, the 5% quantile threshold was taken to determine the significance threshold for each gene ontology term.

#### Independent validation of a major sweep on the X-chromosome

For a large sweep region in the middle of chromosome X, we used additional SNP array genotype data and phenotypic data from two other studies [6, 12] for a more comprehensive examination of this region and its effect on growth.

The samples from [6] comprised 154 GMP, 11 MMP, 4 VPP, 16 European WB and 11 LAR. Pant et al. [12] conducted an F2 cross-breeding experiment in which Duroc and Yorkshire females, respectively, were crossed with Goettingen Minipig males. This study provided X-chromosomal genotypes of 21 GMP males, 6 Duroc and 7 Yorkshire females, 83 F_1_ animals and 454 F_2_animals. All samples were genotyped with the Illumina PorcineSNP60 BeadChip. Size phenotypes for the F_2_animals were also provided.

SNPs within the region of interest, 52 to 61 Mb on the X-chromosome were identified. We used Plink [78] to filter out individuals with more than 90% missing genotypes and SNPs with less than 90% genotyping rate or a minor allele frequency of less than 1%. Under the assumption of no recombination between the haplotypes of the European, Asian and minipig breeds we searched for loci being fixed within a group but showing different states between groups. We then used such informative SNPs to determine the origin of the two haplotypes in the F_2_ animals.

Based on the results of the sequence-based analysis, we hypothesized that the origin of the haplotype in the considered region should affect the body size of F_2_ animals. We therefore classified F_2_ animals in three groups: Homozygous females or hemizygous males carrying the Duroc/ Yorckshire haplotype as first class, heterozygous females as the second class and hemizygous males carrying the minipig haplotype as the third class. These classes were subsequently modeled as a fixed effect nested within sex.

Effects of the minipig haplotype on the four phenotypical traits “shoulder height at slaughter”, “body length at slaughter”, “shoulder height at age of scanning” and “body length at age of scanning” were estimated using proc. “mixed” from SAS 9.4 [84]. The full model was

$$ {y}_{ij k}={B}_i+{S}_j+{b}_1{A}_{ij}+{b}_2{A}_{ij}^2+{H}_k\left({S}_j\right)+{B}_i\times {S}_j+{b}_3\left({B}_i\times {A}_{ij}\right)+{b}_4\left({B}_i\times {A}_{ij}^2\right)+{B}_i\times {H}_k\left({S}_j\right)+{b}_5\left({A}_{ij}\times {H}_k\left({S}_j\right)\right){e}_{ij k} $$

where *y*_*ijk*_ is the dependent variable, *B*_*i*_ is the fixed effect of the breed of the female ancestor in the founder generation (*i* = 1, 2), *S*_*j*_ is the sex, *A*_*ijk*_ is the animal’s age at measurement in days, *H*_*k*_ is the haplotype, either 1 for homozygous females and hemizygous males carrying the large pig haplotype, 2 for heterozygous females and 3 for hemizygous males carrying the minipig haplotype. Each *b*_*l*_(*l* = 1, ..., 5) depicts the linear regression coefficient of the age or the respective interaction of a factor with age. *e*_*ijk*_ is the residual error. The full model was reduced by stepwise backward selection of factors with the highest *p*-values until only significant factors remained.

We employed the R package “rehh” [85] to estimate the extension of the two haplotypes and the decay of linkage disequilibrium around the central position of SNP ‘H3GA0051810’ (56′716’179 bp). Genes within this region were annotated with the Ensembl Pig Gene set 79 [80]. Finally QTL [86] known from former studies located in this region were retrieved from the Pig QTL database (Results not shown).

## Additional files

Additional file 1: **Figure S1.** Multi-Dimensional-Scaling of the distance matrix underlying the phylogenetic tree, based on chromosomes 1, 8 and 13. (DOCX 95 kb)

Additional file 2: **Table S1.** Genome-wide estimated FST values between different contrasts of breed groups for all loci with call-rate ≥ 90%, over diagonal, standard errors below diagonal. (XLSX 10 kb)

Additional file 3: **Table S2.** Genome-wide estimated FST values between different contrasts of breed groups for all loci with call-rate ≥ 90%, over diagonal, standard errors below diagonal. (XLSX 9 kb)

Additional file 4: **Table S3.** Putative selective sweeps with genes contained. (XLSX 12 kb)

Additional file 5: **Table S4.** Potentially overrepresented GO-Terms in selective sweeps. (XLSX 14 kb)

Additional file 6: **Figure S2.** Neighbor-joining tree based on all SNPs in the second part of the selective sweep region on chromosome X between 64 to 96 Mb. (DOCX 61 kb)

Additional file 7: **Table S5.** Overview of SNPs in the region from 52 to 61 Mb on chromosome X, including results of filtering. (XLSX 10 kb)

Additional file 8: **Table S6.** Occurring haplotypes in region 52 to 61 Mb on chromosome X and numbers of carrier animals. (XLSX 10 kb)

Additional file 9: **Table S7.** Genes located in first region of large sweep on chromosome X. (XLSX 9 kb)

Additional file 10: **Table S8.** Overview of sampled breeds and descriptive statistics of the re-sequenced samples. (XLSX 10 kb)

## Abbreviations

BMP: Mini-LEWE; Minischwein Lehnitz-Wendefeld
Chr: Chromosome
CLR: Composite likelihood ratio test
DNA: Deoxyribonucleic acid
DUR: Duroc
ENA: European Nucleotide Archive
GATK: Genome Analysis Toolkit
GMP: Goettingen Minipig
GO: Gene ontology
GQ: Genotype quality
HAM: Hampshire
IBS: Identical by state
JQH: Jiangquhai
LAR: Landrace
LD: Linkage disequilibrium
LPP: Large pig pool
LSD: Least significant difference
LW: Large White
Mb: Mega base pairs, 10^6 base pairs
MEI: Meishan
MMP: Minnesota Minipig
MPP: Minipig pool
PIE: Pietrain
QTL: Quantitative trait loci
SNP: Single nucleotide polymorphism
VCF: Variant call format
VPP: Vietnamese Potbellied Pig
WB FR: Wild boar France
WB JA: Wild boar Japan
WB NC: Wild boar North China
WB NL: Wild boar Netherlands
WB SC: Wild boar South China
WB SW: Wild boar Switzerland
WGS: Whole genome sequencing
WUS: Wuzhishan
XIA: Xiang
