# Supplementary material for: Analysis of porcine body size variation using re-sequencing data of miniature and large pigs
Source: BMC Genomics. 2018 Sep 19;19:687. doi: 10.1186/s12864-018-5009-y (PMC6146782; doi:10.1186/s12864-018-5009-y)
Supplement: Supplementary file 1 — Figure S1. Multi-Dimensional-Scaling of the distance matrix underlying the phylogenetic tree, based on chromosomes 1, 8 and 13. (DOCX 95 kb) [file 12864_2018_5009_MOESM1_ESM.docx]

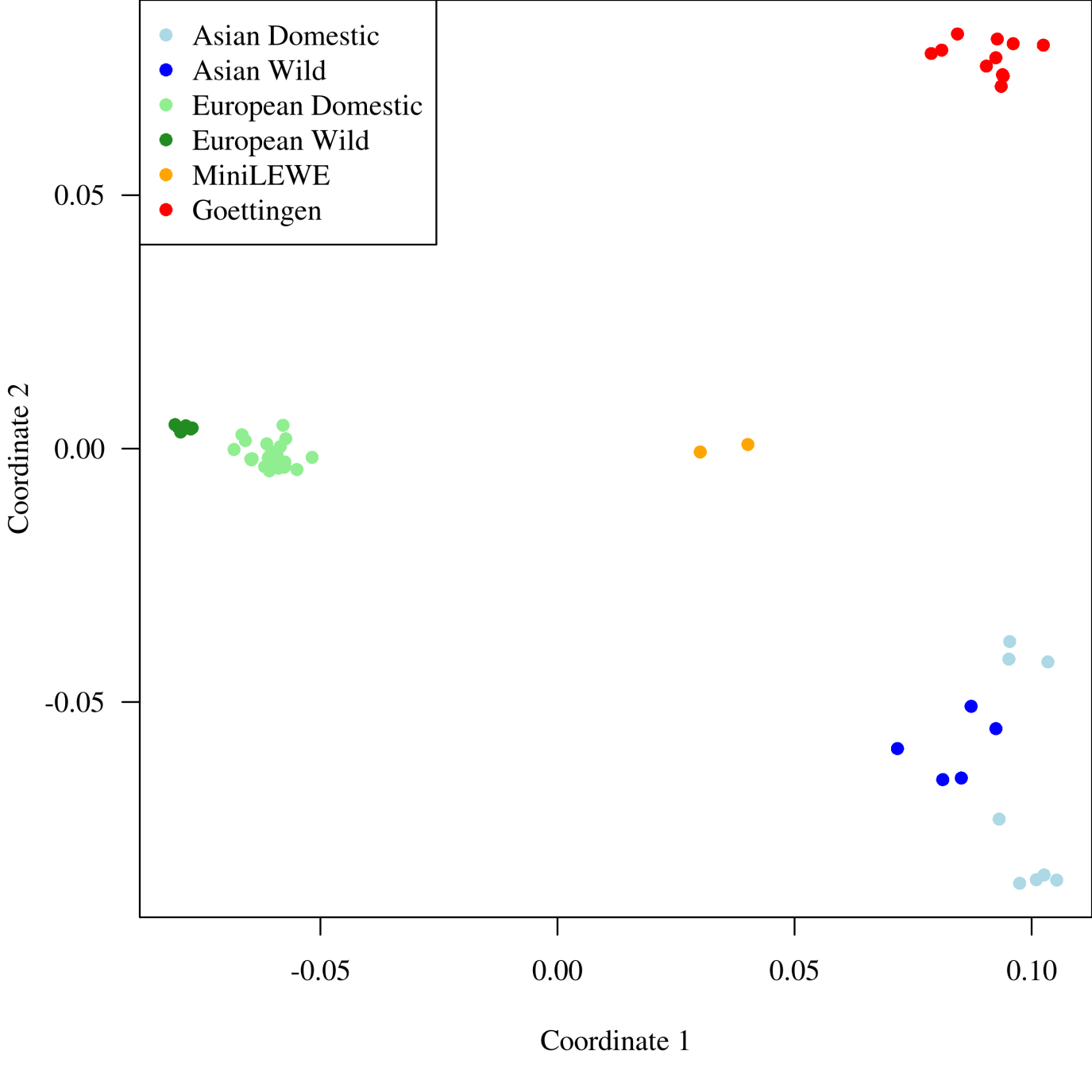


Figure S1: Multi-Dimensional-Scaling of the distance matrix underlying the phylogenetic tree, based on chromosomes 1, 8 and 13.
