# Supplementary material for: Analysis of porcine body size variation using re-sequencing data of miniature and large pigs
Source: BMC Genomics. 2018 Sep 19;19:687. doi: 10.1186/s12864-018-5009-y (PMC6146782; doi:10.1186/s12864-018-5009-y)
Supplement: Supplementary file 6 — Figure S2. Neighbor-joining tree based on all SNPs in the second part of the selective sweep region on chromosome X between 64 to 96 Mb. (DOCX 61 kb) [file 12864_2018_5009_MOESM6_ESM.docx]

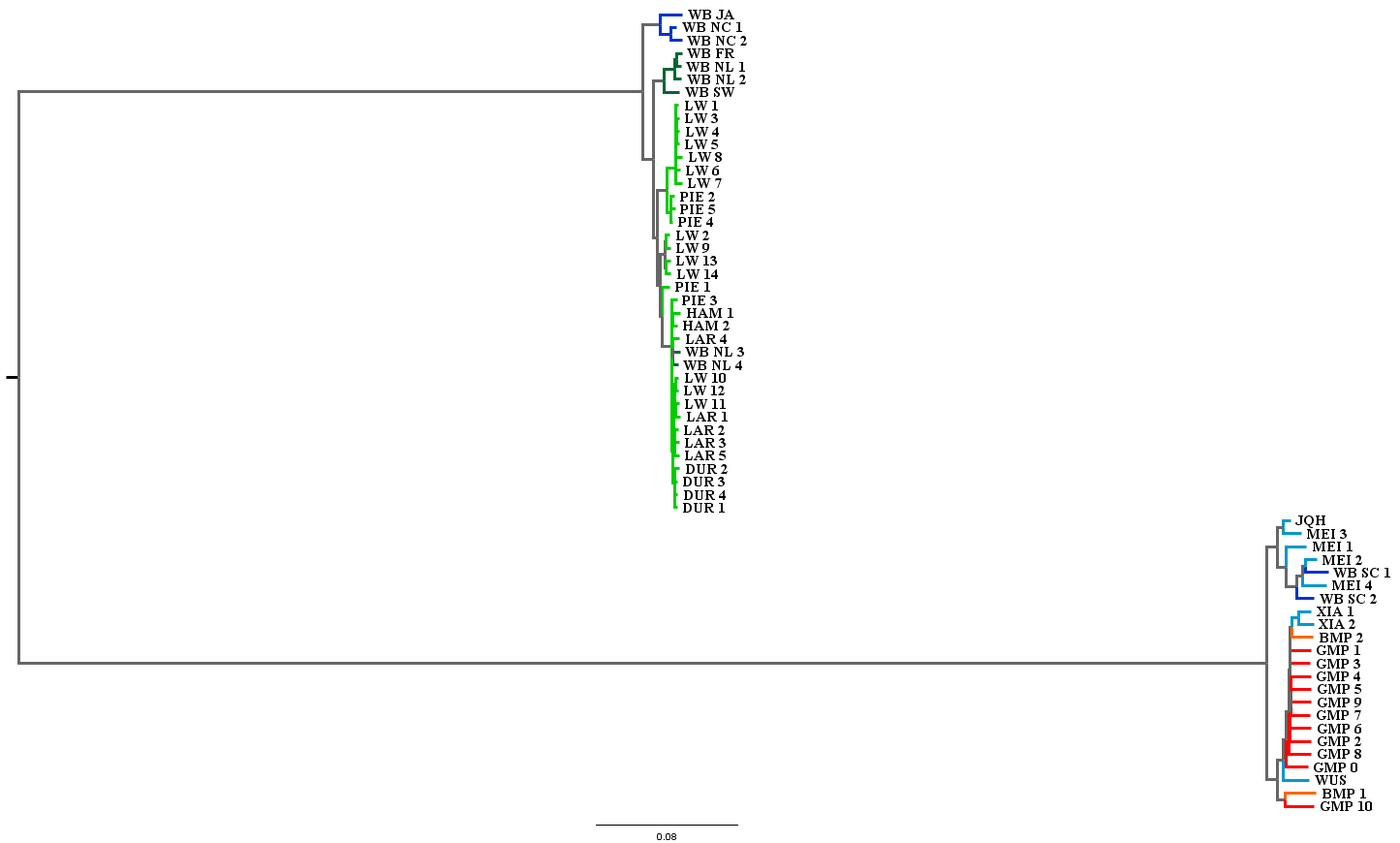


**Figure S2: Neighbor-joining tree based on all SNPs in the second part of the selective sweep region on chromosome X between 64 to 96 Mb.**
